# Single Nucleotide Polymorphism rs6942067 Is a Risk Factor in Young and in Non-Smoking Patients with HPV Negative Head and Neck Squamous Cell Carcinoma

**DOI:** 10.3390/cancers12010055

**Published:** 2019-12-24

**Authors:** Guillaume B. Cardin, Monique Bernard, Houda Bahig, Phuc Felix Nguyen-Tan, Olivier Ballivy, Edith Filion, Denis Soulieres, Pierre Philouze, Tareck Ayad, Louis Guertin, Eric Bissada, Francis Rodier, Apostolos Christopoulos

**Affiliations:** 1 Centre de Recherche du Centre Hospitalier de l’Université de Montréal, Montréal, QC H2X 0A9, Canada; cardinguill@gmail.com (G.B.C.); bernardmonique07@gmail.com (M.B.); houdabahig@gmail.com (H.B.); tareck.ayad@umontreal.ca (T.A.); rodierf@mac.com (F.R.); 2 Institut du Cancer de Montréal, Montréal, QC H2X 0A9, Canada; 3 Department of Radiation Oncology, Centre hospitalier de l’Université de Montréal, Montréal, QC H2X 3E4, Canada; felixnguyentan@gmail.com (P.F.N.-T.); olivier.ballivy.chum@ssss.gouv.qc.ca (O.B.); edith.filion@gmail.com (E.F.); 4 Department of Medicine, Service of Hemato-Oncology, Centre Hospitalier de l’Université de Montréal, Montréal, QC H2X 3E4, Canada; denis.soulieres@umontreal.ca; 5 Otolaryngology-Head and Neck Surgery Service, Centre Hospitalier de l’Université de Montréal, Montréal, QC H2X 3E4, Canada; pierre.philouze@chu-lyon.fr (P.P.); guertinorl@gmail.com (L.G.); ericbissada@gmail.com (E.B.); 6 Département de Radiologie, Radio-Oncologie et Médecine Nucléaire, Université de Montréal, Montréal, QC H2X 3E4, Canada

**Keywords:** head and neck squamous cell carcinoma, rs6942067, DCBLD1, cancer susceptibility genes, tobacco, human papillomavirus

## Abstract

Genetic factors behind the increasing incidence of human papillomavirus (HPV) negative head and neck squamous cell carcinoma (HNSCC) in young non-smokers are suspected, but have not been identified. Recently, rs6942067, a single nucleotide polymorphism (SNP) located upstream of the DCBLD1 gene, was found associated with non-smoking lung adenocarcinoma. To validate if this SNP is also implicated in HNSCC, participants of The Cancer Genome Atlas HNSCC cohort were investigated for rs6942067 status, associated DCBLD1 expression, and clinical characteristics. Occurrence of the rs6942067 GG genotype is significantly higher in young and in HPV negative non-smoking HNSCC than in other HNSCC. Additionally, rs6942067 GG is associated with higher DCBLD1 expression in HNSCC and patients with high DCBLD1 expression have a worse overall survival at three years, both in univariate and multivariate analysis. Furthermore, high DCBLD1 expression is associated with activation of the integrin signaling pathway and its phosphorylation with EGFR and MET. Collectively, these findings suggest that DCBLD1 plays a critical role in HNSCC and demonstrate an association between rs6942067 and clinical characteristics of young age and HPV negative non-smoking status in HNSCC patients.

## 1. Introduction

Head and neck squamous cell carcinoma (HNSCC) is a group of cancers occurring in the oral cavity, oropharynx, hypopharynx, and larynx, which accounts for over 650,000 new cases and 330,000 deaths annually worldwide [1]. HNSCC patients usually present classic risk factors, which are tobacco and alcohol use and/or human papillomavirus (HPV) infection [2,3,4]. While increased occurrence of HPV has been implicated in young patients developing HNSCC, there has also been a parallel increasing incidence of young patients with HNSCC with no known risk factors [5,6,7,8]. Most typically, these latter patients are characterized as young non-smokers, most often females, presenting with HPV negative disease, with the most frequent subsite being the oral tongue [5,6,7,9,10,11,12,13,14]. Over the last 45 years, many studies reported that those young patients developing HNSCC, especially in the oral tongue, had a more aggressive disease and a worse prognosis than older patients, although some studies also reported no significant difference [15,16,17,18,19,20,21,22]. Family history of cancer in young relatives has been associated with head and neck cancer; therefore, suggesting the presence of a genetic factor in these patients [9]. Such genetic factor is yet to be identified.

Whole exome analysis of young non-smoking HNSCC patients compared to older smoking patients has been studied but exomes were similar in both groups [23]. Whole genome analysis looking at single nucleotide polymorphisms (SNPs) was never studied in HNSCC, partly because of the difficulty to build a large enough cohort to obtain sufficient statistical power. On the other hand, it has been analyzed in non-smoking lung adenocarcinoma on a cohort of 6609 never-smoking and 7457 control patients, finding a SNP-heavy region upstream of the DCBLD1 gene, although no relationship with age was reported [24,25]. It was later shown in vitro that it is one specific SNP from this region, rs6942067, which causes an upregulation of the DCBLD1 gene, and the association between rs6942067 and DCBLD1 overexpression was corroborated in vivo in lung and thyroid tissue [26]. Whether rs6942067 or DCBLD1 would be implied in non-smoking or other HNSCC was never studied.

DCBLD1 is a transmembrane protein with domain structures similar to that of neuropilins [27]. DCBLD1 has been associated with lung adenocarcinoma and glioma [24,25,26,27]. DCBLD1 remains largely uncharacterized and not many other human studies have been published. A functional study identified YxxP motifs in DCBLD1, which are phosphorylated by Src family kinases and Abl, and promote the binding of CRK adaptor proteins, although the implications are unknown [28]. Although DCBLD1 was never studied in HNSCC, DCBLD2 (paralog with DCBLD1) has been shown to be a bad prognostic marker in HNSCC when co-activated with EGFR [29].

While DCBLD1’s function remains unclear, it seems relevant in specific cancer settings. The purpose of this study is to evaluate the presence and clinical relevance of rs6942067 and DCBLD1 specifically in the young and HPV negative non-smoking patients with HNSCC.

## 2. Results

### 2.1. rs6942067 Status in HPV Negative Non-Smoker HNSCC and Young HNSCC

**Figure 1:**
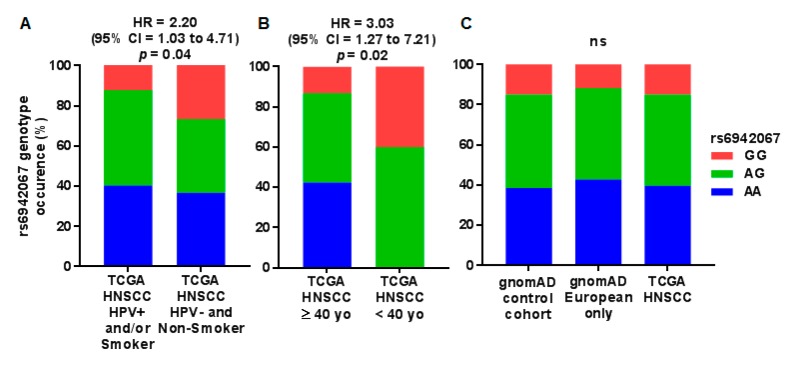
rs6942067 genotype status in the Cancer TCGA HNSCC cohort and the gnomAD cohort. Comparison of rs6942067 genotype for (**A**) HPV positive and/or smoking patients (*n* = 124) and non-smoker and HPV negative patients (*n* = 30) of the TCGA HNSCC cohort; (**B**) ≥40 years old (*n* = 144) and <40 years old (*n* = 10) patients of the TCGA HNSCC cohort; and (**C**) all participants of the gnomAD cohort (*n* = 15,654), European participant of the gnomAD cohort (*n* = 9431), and patients of the TCGA HNSCC cohort. Only 154 patients had whole genome sequencing data for the TCGA HNSCC cohort. Relative risk and *p* values are for a Pearson’s chi-square test and a recessive model of penetrance.

To evaluate if rs6942067 could explain in part the occurrence of HPV negative non-smoker HNSCC, the Cancer Genome Atlas (TCGA) HNSCC cohort (154 participants with whole genome sequencing data, rs6942067 AA = 61, AG = 70, GG = 23) was used as the investigational population. Patients from the TCGA HNSCC cohort were stratified in an HPV positive and/or smoker group (rs6942067 AA = 50, AG = 59, GG = 15) and an HPV negative non-smoker group (rs6942067 AA = 11, AG = 11, GG = 8). The HPV negative non-smoker group showed higher occurrence of the homozygous GG genotype in comparison with other HNSCC (hazard ratio (HR) = 2.20, 95% confidence interval (CI) = 1.03 to 4.71), suggesting a recessive effect (Figure 1A).

To test if rs6942067 could also explain the occurrence of HNSCC in a younger population, rs6942067 status was determined for HNSCC in a defined young population (<40 years old, rs6942067 AA = 0, AG = 6, GG = 4) versus older HNSCC (≥40 years old, rs6942067 AA = 61, AG = 64, GG = 20) in the TCGA HNSCC cohort (Figure 1B). The occurrence of young HNSCC cases was small but their genotype distribution was significantly different from older HNSCC cases (HR = 3.03, 95% CI = 1.27 to 7.21).

Distribution of rs6942067 genotype was also investigated regarding alcohol usage for the 67 patients from the TCGA HNSCC cohort which had both alcohol history and whole genome sequencing data. They were stratified in an HPV positive and/or drinker group (rs6942067 AA = 20, AG = 23, GG = 4) and an HPV negative non-drinker group (rs6942067 AA = 5, AG = 11, GG =4). The HPV negative non-drinker group showed non-significant higher occurrence of the homozygous GG genotype in comparison with other HNSCC (HR = 2.35, 95% CI = 0.65 to 8.48) (Figure S1).

It was then validated that the HNSCC TCGA cohort had a similar rs6942067 genotype distribution then the healthy population. The gnomAD cohort was used as the healthy control group (mostly from case-control studies, rs6942067 AA = 6031, AG = 7282, GG = 2341). Since the HNSCC TCGA cohort is mostly Caucasians, European participants of the gnomAD cohort (rs6942067 AA = 4037, AG = 4290, GG = 1104) were also looked at, specifically, as a generally Caucasian cohort. No significant difference was observed between the three groups (Figure 1C) and the HNSCC TCGA cohort showed a phenotype distribution much alike the gnomAD cohort.

Clinical features were different according to rs6942067 genotype in the HNSCC TCGA cohort (Table 1). There was no significant difference for age group, gender, anatomical subsite, smoking status, HPV status, tumor size, and nodal status. *p* values were calculated by a two-sided Pearson’s chi-square test and corrected for false discovery rate (FDR) using the Benjamini–Hochberg procedure. The absence of independent significance for HPV and smoking status suggests that it is the complete absence of HNSCC risk factor which is associated with the rs6942067 GG genotype.

### 2.2. DCBLD1 Association with rs6942067

**Figure 2:**
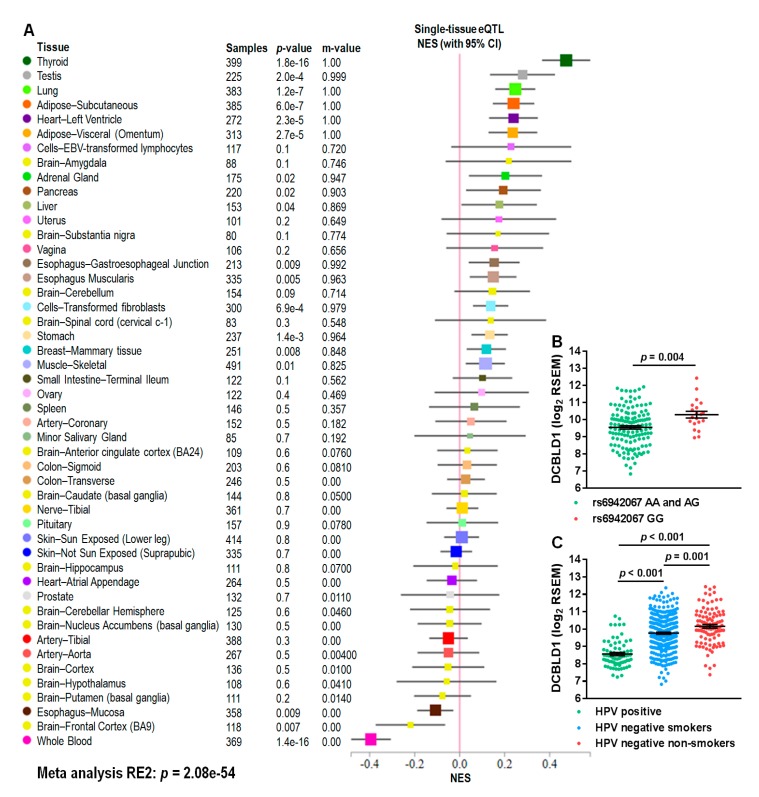
DCBLD1 gene expression, rs6942067, and HNSCC risk factors. (**A**) Multi-tissue expression quantitative trait locus analysis of rs6942067 and DCBLD1 using the GTEx project and the Han and Eskin’s random effects model (RE2). (**B**,**C**) Comparison of DCBLD1 gene expression for (**B**) rs6942067 AA and AG (*n* = 135) or rs6940267 GG (*n* = 20) and (**C**) HPV positive (green, *n* = 71), HPV negative smokers (blue, *n* = 334), or HPV negative non-smokers (red, *n* = 101) patients of the TCGA HNSCC cohort. Mean and standard error are shown.

To evaluate the global effect of rs6942067 on DCBLD1 gene expression, we used multi-tissue expression quantitative trait locus analysis from the Genotype-Tissue Expression (GTEx) project. Meta-analysis using the Han and Eskin’s random effects model (RE2) [30] shows a significant human multi-tissue association (RE2: *p* = 2.1 × 10^−54^) between rs6942067 and DCBLD1 expression (Figure 2A). This analysis shows that in most normal human tissues, the rs6942067 G allele is associated with an upregulation of DCBLD1 expression.

More specifically in the TCGA HNSCC cohort, DCBLD1 expression is upregulated in the rs6942067 GG patients of that cohort in comparison to other genotype (*p* = 0.004) (Figure 2B). Additionally, DCBLD1 expression is upregulated in the HPV negative non-smoker patients subgroup, in comparison to HPV positive (*p* < 0.001) and HPV negative smoker patients (*p* = 0.001) (Figure 2C), thus strengthening the hypothesis that it is the complete absence of HNSCC risk factor which is associated with the rs6942067 GG genotype.

### 2.3. DCBLD1 Association with Clinical Features and Overall Survival

**Figure 3:**
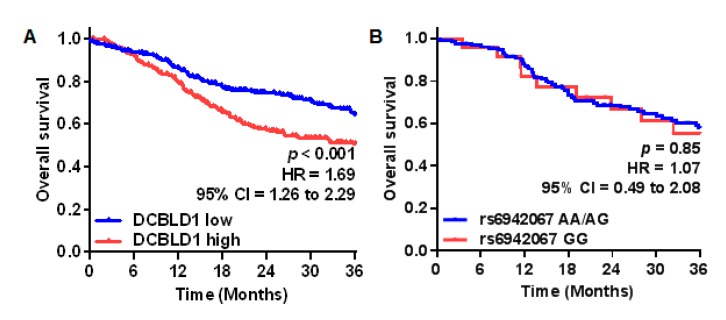
DCBLD1 gene expression, rs6942067, and HNSCC overall survival comparison for HNSCC patients of the TCGA cohort using the Kaplan–Meier curve estimation for (**A**) tumor low (blue, *n* = 258) and high (red, *n* = 259) DCBLD1 gene expression; and for (**B**) rs6942067 AA and AG (blue, *n* = 131) versus rs6942067 GG (red, *n* = 23). *p* values were calculated using a two-sided log-rank test.

HNSCC patients of the TCGA cohort were stratified in a DCBLD1-low (*n* = 258) and a DCBLD1-high (*n* = 259) group using the expression median. The impact of DCBLD1 expression and rs6942067 genotype on patients’ overall survival was then evaluated on the TCGA HNSCC cohort (Figure 3).

Univariate Kaplan–Meier analysis showed that high DCBLD1 gene expression was significantly associated to a worse overall survival at three years (HR = 1.69, 95% CI = 1.26 to 2.29), but no difference in overall survival was observed for the rs6942067 GG genotype.

Clinical characteristics were compared between the DCBLD1-high and DCBLD1-low groups (Table 2). The DCBLD1-high patients had lower occurrence of HPV (*p* < 0.001), were more often female (*p* = 0.02), and had a different anatomical subsite distribution (*p* < 0.001), with 2.4 times more occurrence of oral tongue cancer. There were no significant difference for age groups, smoking status, tumor size, and nodal status.

Since DCBLD1 expression is strongly associated to HPV status and HPV is an important prognostic factor for oropharyngeal SCC [31], we evaluated the independence of DCBLD1 as a prognostic marker. Univariate and multivariate Cox proportional hazards analysis of overall survival were performed on the HNSCC TCGA cohort (Table 3). Multivariate Cox proportional hazards analysis of overall survival showed that DCBLD1 expression is an independent prognostic marker (HR = 2.69, 95% CI = 1.09 to 6.58) along with HPV status, nodal status, and age. Those factors were also significantly predictive of overall survival in the univariate analysis. Gender and anatomical subsite were significantly predictive of overall survival in the univariate analysis but not in the multivariate analysis. DCBLD1 expression and age were analyzed as numerical variables. Assumption of proportionality was verified by testing that the interactions between survival time and covariates were statistically not significant.

### 2.4. Bioinformatical Analysis of DCBLD1 Function

**Figure 4:**
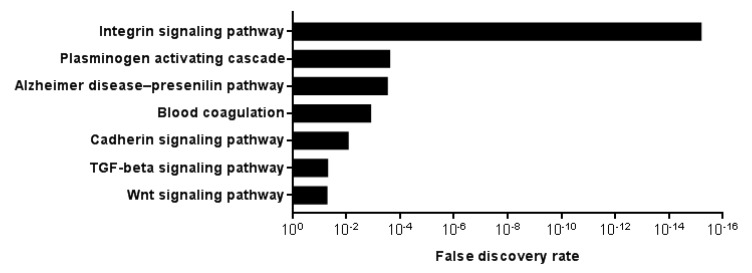
Significantly enriched PANTHER pathways in the higher (*n* = 25) versus lower (*n* = 25) DCBLD1 expressing participants from the HNSCC TCGA cohort. *p* values for significance cut-off was calculated using Bonferroni correction. No pathway was significantly enriched in the lower DCBLD1 participants.

To get insight into DCBLD1 function in HNSCC, we analyzed RNAseq data from the 25 highest and 25 lowest DCBLD1-expressing patients from the HNSCC TCGA cohort, to identify genes that were significantly differentially expressed in the two groups using Bonferroni correction. Those two groups represent the top and bottom 5% of the 517 participants with RNAseq data. Upregulated and downregulated genes were then independently tested for gene ontology using the PANTHER pathways (Figure 4). For significantly enriched pathways in the DCBLD1-high group, the integrin signaling pathway especially stood out with an FDR of 6.08 × 10^−16^. Other significantly enriched pathways were the plasminogen activating cascade, the Alzheimer disease–presenilin pathway, blood coagulation, the cadherin signaling pathway, the transforming growth factor beta (TGF–beta) signaling pathway and the Wnt signaling pathway. There was no significantly enriched pathway in the DCBLD1-low group. We also performed this analysis using the 50 highest and 50 lowest DCBLD1 expressing patients (Figure S2). Results were similar to the previous analysis, although significance was higher in all pathways and four other pathways related to cell function became significant.

**Figure 5:**
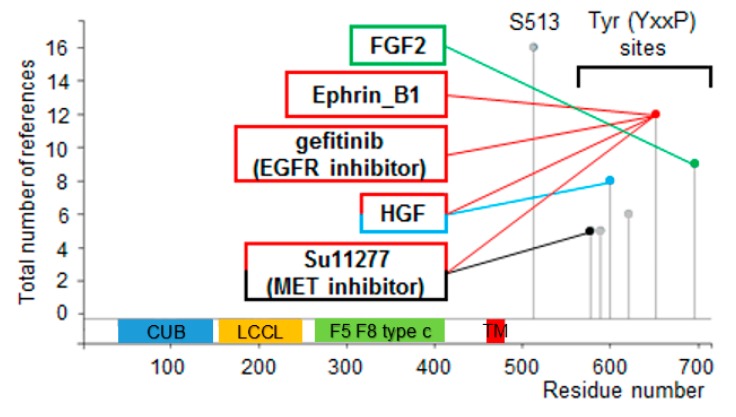
Phosphoproteomic bioinformatical analysis of the DCBLD1 protein. PhosphoSitePlus^®^ analysis shows one frequently detected S513 phosphorylation site of unknown function and six YxxP sites. Four of the six sites are linked with three different receptor tyrosine kinases.

Protein modifications and their regulation are linked to function. PhosphoSitePlus^®^ analysis on the DCBLD1 protein reveals two types of modifications on DCBLD1: An S513 modification, which is highly present but not associated with any specific condition; and six YxxP sites (Figure 5). Those YxxP sites are the same ones which were previously associated with the Src family kinases, Abl, and CRK adaptor proteins [28]. Our analysis showed that two important receptor tyrosine kinases are also linked to these YxxP sites—EGFR and MET—which are, respectively, ligands for EGF and HGF.

## 3. Discussion

The reason why we observe an increasing incidence of young patients with HNSCC with no known risk factors has been elusive for decades. In this study, we report a SNP (rs6942067) which is associated with HNSCC in non-smoker HPV negative patients and also in young (<40 years old) patients. It is important to state that a large corroboration cohort will be needed before drawing definitive conclusions on the associations presented here in HNSCC. This SNP had been previously identified in a susceptibility loci at 6q22.2 for patients with higher risk of developing never-smoking lung adenocarcinoma [24]. It was shown in vitro that rs6942067 is the SNP causing an upregulation of the DCBLD1 gene [26]. We validated this association between rs6942067 and DCBLD1 in HNSCC, but, while we show that DCBLD1 is a prognosis factor in HNSCC, interestingly rs6942067 itself is not associated with HNSCC overall survival. This is somewhat expected given that other unknown factors probably affect DCBLD1 gene expression, as there are many patients with high DCBLD1 expression in the rs6942067 AA and AG group in Figure 2b. Additionally, rs6942067 GG occurrence in the healthy population is too high to explain on its own why some young patients with no classical HNSCC risk factors develop HNSCC. It is possible that additional unknown factors are synergizing with rs6942067 in young and non-smokers with HPV negative HNSCC, and future studies will be necessary to answer this question.

It has been suggested in many studies that young patients with no classical HNSCC risk factors have a worse prognosis than other patients. Whether DCBLD1 overexpression would be the explanation for that reported worse prognosis is unknown. Interestingly, DCBLD1-high patients have a higher occurrence of oral tongue cancer and are more often female, all characteristics which were previously described in young patients with HNSCC with no known risk factors. Our gene ontology analysis suggests many pathways are associated with DCBLD1 expression but the integrin signaling pathway especially stood out with an FDR of 6.08 × 10^−16^. This pathway is triggered when integrins in the cell membrane bind to extracellular matrix components, and downstream events include actin reorganization and activation of the MAPK signaling cascade [32,33,34,35,36,37]. In HNSCC, the integrin signaling pathway has been associated with progression, stemness, and resistance to radiotherapy [38,39,40].

Our PhosphoSitePlus^®^ analysis showed that many receptor tyrosine kinases are linked to DCBLD1 phosphorylation. Of special interest are MET and its ligand (HGF) reported for Y578, Y600, and Y652 regulation, and EGFR reported for Y652 regulation. MET has been reported as a poor prognosis marker in HNSCC in many studies [41,42,43,44,45]. EGFR is an important protein in HNSCC as it is associated with a worse outcome in the context of overexpression and is the target of Cetuximab [46,47,48]. The fact that DCBLD1 is a strong prognosis factor in HNSCC and experimentally associated with EGFR and MET suggests that DCBLD1 may play a critical role in HNSCC. Interestingly, DCBLD2, which is paralog to DCBLD1, has also been shown to be interacting with receptor tyrosine kinases such as EGFR, VEGFR, and INSR [28,49,50]. In the never-smoking lung adenocarcinoma study, the DCBLD1 susceptibility locus was associated with EGFR mutations in exons 19 and 21 (in the tyrosine kinase encoding region) [24,25].

The current study has limitations that must be stated. First, there is a need for a larger confirmation cohort to corroborate our results before drawing definitive conclusions, as this study was limited by the number of participants, especially for the analysis of the impact of alcohol use on rs6942067 genotype distribution, as only 67 participants of the TCGA HNSCC cohort had documented alcohol history and whole genome sequencing data. Second, it would be interesting to evaluate the occurrence of HNSCC patients that are both under 40 years old and HPV negative non-smokers. Sadly, there were only three such patients with whole genome sequencing data available in the TCGA HNSCC cohort and that analysis was; therefore, impossible.

It is important to acknowledge that the prognostic value of DCBLD1 detailed here is made without evaluation of treatment applied to the studied populations, which is itself dependent on the disease state. Since treatment is standard for most (surgery, radiation therapy, and chemoradiation therapy), predictive value of DCBLD1 could be evaluated by studying an interaction in statistical terms. Such evaluation would be highly interesting, but will have to be conducted from samples of patients treated in randomized trials to determine the predictive value of this gene to define therapy.

## 4. Materials and Methods 

### 4.1. Study Cohorts

This study involved 525 participants of the TGCA HNSCC cohort (TCGA, Provisional) [51]. Three participants from this cohort with DCBLD1 gene mutations were excluded from this study, as DCBLD1 mutations are too rare in this cohort (3 cases) to reach meaningful conclusions. As a reference cohort for rs6942067 status, we used the 15,654 participants of the Genome Aggregation Database (gnomAD) from the GTEx project [52]. Data obtained from the GTEx Portal and dbGaP Accession phs000424.v7.p2 on 22 May 2019 were used to evaluate rs6942067 global effect on DCBLD1 expression. The TCGA overall survival and gene expression data were extracted using cBioPortal for Cancer Genomics (www.cbioportal.org) [53,54]. Clinical characteristics of the TCGA cohort were extracted from FirehoseR (gdac.broadinstitute.org) [55]. Raw data used for these analysis is available in Table S1 (TCGA) and Table S2 (gnomAD). Access to controlled access data of whole genome sequencing was done though project #19174 (OMB 0925-0670).

### 4.2. Clinical Characteristics

HPV status of the TCGA HNSCC cohort was reported in a previous study [56]. Lifelong non-smokers and current previous smokers for over 15 years with less than 10 pack-year (PY) smoked were classified as non-smokers. Current smokers and other previous smokers were considered as smokers. For young patient age cut-off, <40 years old was used for rs6942067 occurrence, based on previous studies for young HNSCC patients with no classical risk factors [11,21,57,58,59]. For anatomical subsites, oral tongue was separated from other oral cavity cancers to evaluate previously reported higher occurrence of oral tongue cancer for young non-smoking HPV negative HNSCC. Tumor size (pT) was divided between small (≤T2, ≤4.0 cm) and large (≥T3, >4.0 cm) tumors. Nodal status was divided between negative (N0) and positive lymph node status (N+). For alcohol use, patients with an average of ≥1 drink per day were considered drinkers, while patients with an average of <1 drink per day were considered non-drinkers. The rs6942067A/G genotype of 154 participants of the TCGA HNSCC cohort was determined using whole genome sequencing data. Other participants of this cohort had no available information regarding their rs6942067A/G genotype. This SNP is located on chr6:117,464,533 of GRCh38.p12.

### 4.3. Bioinformatical Analysis

Multi-tissue expression quantitative trait locus analysis was done using the GTEx Portal [52]. Gene ontology analysis of genes upregulated for the 25 participants of the TCGA HNSCC cohort with the higher versus lower DCBLD1 gene expression was performed using the PANTHER pathway database to evaluate pathway enrichment [60,61,62]. PhosphoSitePlus^®^ is a bioinformatical tool that finds reports of protein modifications in low and high-throughput phosphoproteomic data sources, and then searches for experimentally verified regulation by specific treatments or other binomial stratifications [63]. We used a cut-off of five reports of protein modification for this analysis.

### 4.4. Statistics

Genotype frequencies and variations in clinical characteristics were analyzed using a Pearson’s chi-square test. Tukey–Kramer was used for gene expression analysis. Kaplan–Meier curves were assessed using log-rank *p* values. Analysis of clinical characteristics was corrected for FDR using the Benjamini–Hochberg procedure. A multivariate Cox proportional hazards analysis was used to evaluate hazard ratios and to perform the multivariate survival prediction model. Assumption of proportionality for this analysis was verified by testing that the interactions between survival time and covariates were statistically not significant.

Throughout the analyses, tests of statistical significance are two-sided and *p* values less than 0.05 corrected for multiple testing were considered statistically significant. Statistical analysis were performed using JMP 12.0.1 statistical software (SAS Institute Inc., Cary, NC, United States).

## 5. Conclusions

In summary, our study demonstrates an association between rs6942067 and clinical characteristics of young age and HPV negative non-smoking status in HNSCC patients. This study also associates high DCBLD1 expression with a worse prognosis and the integrin signaling pathways in HNSCC. We also showed an association of DCBLD1 phosphorylation with EGFR and MET.

## Figures and Tables

**Table 1:** Clinical features of the TCGA HNSCC cohort stratified by rs6942067 status.

Characteristics	rs6942067 (AA/AG)	rs6942067 (GG)	*p*
**Total**	131	23	
**Age group, *n* (%)**			0.12
≥40 years old	125 (95.4)	19 (82.6)	
<40 years old	6 (4.6)	4 (17.4)	
**Gender, *n* (%)**			0.43
Female	38 (29.0)	4 (17.4)	
Male	93 (71.0)	19 (82.6)	
**Anatomical subsite, *n* (%)**			0.88
Oral tongue	47 (35.9)	8 (34.8)	
Other oral cavity	42 (32.1)	6 (26.1)	
Oropharynx	23 (17.6)	4 (17.4)	
Larynx and hypopharynx	19 (14.5)	5 (21.7)	
**Smoking status, *n* (%)**			0.14
Current/previous ≥ 10 PY ^1^	99 (75.6)	13 (56.5)	
Never/previous < 10 PY ^1^	32 (24.4)	10 (43.5)	
**HPV, *n* (%)**			0.59
Positive	33 (25.2)	4 (17.4)	
Negative	98 (74.8)	19 (82.6)	
**Tumor size, *n* (%)**			0.88
T1-2	53 (40.5)	9 (39.1)	
T3+	77 (58.8)	14 (60.9)	
Tx, unknown	1 (0.8)	0 (0)	
**Nodal status, *n* (%)**			0.12
N0	64 (48.9)	6 (26.1)	
N+	64 (48.9)	17 (73.9)	
Nx, unknown	2 (1.5)	0 (0)	

^1^ Pack-year.

**Table 2:** Clinical features of the TCGA HNSCC cohort stratified by DCBLD1 gene expression.

Characteristics	DCBLD1-High	DCBLD1-Low	*p*
**Total**	258	259	
**Age group, *n* (%)**			0.74
≥40 years old	250 (96.9)	248 (95.8)	
<40 years old	8 (3.1)	10 (3.9)	
unknown	0 (0)	1 (0.4)	
**Gender, *n* (%)**			0.02
Female	55 (21.3)	81 (31.3)	
Male	203 (78.7)	178 (68.7)	
**Anatomical subsite, *n* (%)**			<0.001
Oral tongue	38 (14.7)	92 (35.5)	
Other oral cavity	85 (32.9)	98 (37.8)	
Oropharynx	60 (23.3)	19 (7.3)	
Larynx and hypopharynx	75 (29.1)	50 (19.3)	
**Smoking status, *n* (%)**			0.15
Current/previous ≥ 10 PY ^1^	198 (76.7)	182 (70.3)	
Never/previous < 10 PY ^1^	54 (20.9)	71 (27.4)	
Unknown	6 (2.3)	6 (2.3)	
**HPV, *n* (%)**			<0.001
Positive	64 (24.8)	7 (2.7)	
Negative	194 (75.2)	252 (97.3)	
**Tumor size, *n* (%)**			0.67
T1-2	97 (37.6)	89 (34.3)	
T3+	154 (59.7)	161 (62.2)	
Tx, unknown	7 (2.7)	9 (3.5)	
**Nodal status, *n* (%)**			0.75
N0	119 (46.1)	122 (47.1)	
N+	129 (50.0)	125 (48.3)	
Nx, unknown	10 (3.9)	12 (4.6)	

^1^ Pack-year.

**Table 3:** Multivariate Cox proportional hazards analysis of overall survival in the TCGA HNSCC cohort.

Variable	Univariate Analysis		Multivariate Analysis	
	HR (95% CI)	*p*	HR (95% CI)	*p*
**HPV**		<0.001		0.001
Negative	Reference		Reference	
Positive	0.27 (0.13 to 0.50)		0.28 (0.11 to 0.62)	
**Nodal status**		0.04		0.006
N0	Reference		Reference	
N+	1.35 (1.01 to 1.82)		1.56 (1.13 to 2.15)	
**Age**, years old (range) ^1^	3.48 (1.38 to 8.86)	0.008	3.44 (1.22 to 9.91)	0.02
**DCBLD1**, log_2_ RSEM (range) ^1^	3.97 (1.87 to 8.43)	<0.001	2.69 (1.09 to 6.58)	0.03
**Smoking status**		0.30		0.24
Never/previous < 10 PY ^2^	Reference		Reference	
Current/previous ≥ 10 PY ^2^	1.21 (0.86 to 1.74)		1.26 (0.86 to 1.89)	
**Tumor size**		0.09		0.29
T1-2	Reference		Reference	
T3+	1.31 (0.96 to 1.79)		1.21 (0.85 to 1.73)	
**Gender**		0.02		0.43
Female	Reference		Reference	
Male	0.70 (0.51 to 0.95)		0.87 (0.62 to 1.24)	
**Anatomical subsite**		0.01		0.61
Oropharynx	Reference		Reference	
Oral tongue	2.00 (1.16 to 3.64)		0.83 (0.44 to 1.67)	
Other oral cavity	2.23 (1.35 to 3.97)		0.92 (0.50 to 1.78)	
Larynx and hypopharynx	1.70 (0.98 to 3.12)		0.71 (0.37 to 1.44)	

^1^ HR > 1 for numerical variables means that higher expression is associated with a worse overall survival. ^2^ Pack-year.

## Supplementary Materials

The following are available online at https://www.mdpi.com/2072-6694/12/1/55/s1, Figure S1: rs6942067 genotype status in the TCGA HNSCC cohort for HPV positive and/or drinking patients (*n* = 47) and HPV negative non-drinker patients (*n* = 20), Figure S2: Significantly enriched PANTHER pathways in the higher (*n* = 50) versus lower (*n* = 50) DCBLD1 expressing participants from the HNSCC TCGA cohort, Table S1: Raw data from the TCGA cohort, Table S2: Raw data from the gnomAD cohort.
