# Supplementary material for: Single Nucleotide Polymorphism rs6942067 Is a Risk Factor in Young and in Non-Smoking Patients with HPV Negative Head and Neck Squamous Cell Carcinoma
Source: Cancers (Basel). 2019 Dec 24;12(1):55. doi: 10.3390/cancers12010055 (PMC7017251; doi:10.3390/cancers12010055)
Supplement: Supplementary file 1 [file cancers-12-00055-s001.zip › Figure S1.pdf]

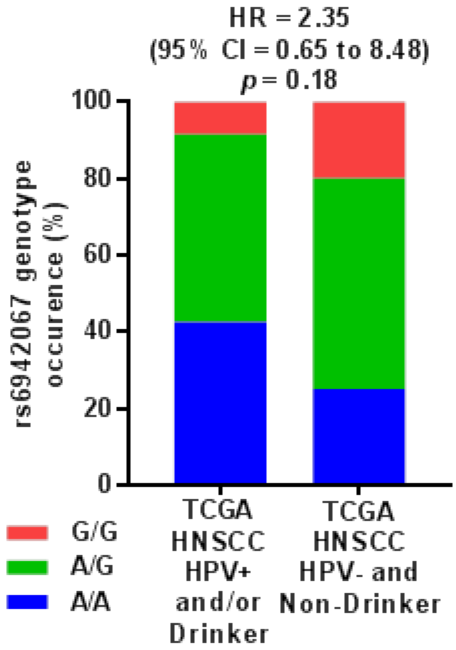

Figure S1. rs6942067 genotype status in the TCGA HNSCC cohort for HPV positive and/or drinking patients (n = 47) and HPV negative non-drinker patients (n = 20). Patients with an average of ≥1 drink per day were considered drinkers while patients with an average of <1 drink per day were considered non-drinkers. Only 67 patients had whole genome sequencing data for the TCGA HNSCC cohort. HR and p values are for a Pearson's chi-square test and a recessive model of penetrance.
