# Supplementary material for: Single Nucleotide Polymorphism rs6942067 Is a Risk Factor in Young and in Non-Smoking Patients with HPV Negative Head and Neck Squamous Cell Carcinoma
Source: Cancers (Basel). 2019 Dec 24;12(1):55. doi: 10.3390/cancers12010055 (PMC7017251; doi:10.3390/cancers12010055)
Supplement: Supplementary file 1 [file cancers-12-00055-s001.zip › Figure S2.pdf]

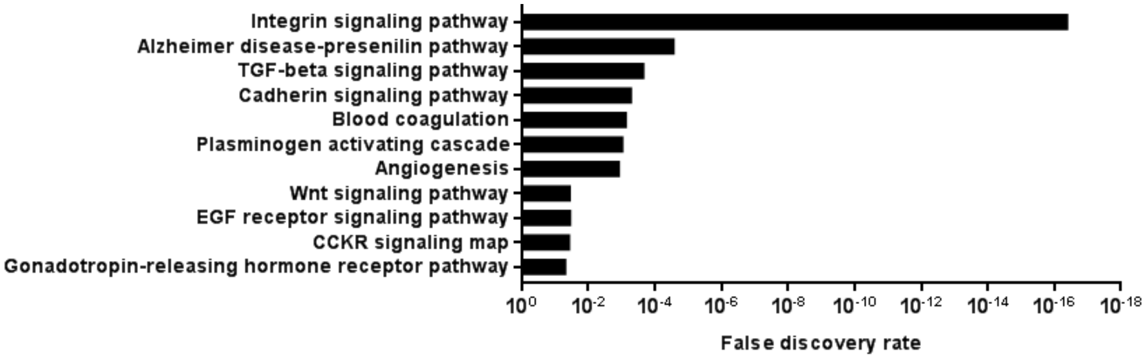

Figure S2. Significantly enriched PANTHER pathways in the higher (n=50) versus lower (n=50) DCBLD1 expressing participants from the HNSCC TCGA cohort. P values for significance cut-off was calculated using Bonferonni correction. No pathway was significantly enriched in the lower DCBLD1 participants.
